# Light and Hormones in Seasonal Regulation of Reproduction and Mood

**DOI:** 10.1210/endocr/bqaa130

**Published:** 2020-08-01

**Authors:** Junfeng Chen, Kousuke Okimura, Takashi Yoshimura

**Affiliations:** 1 Institute of Transformative Bio-Molecules (WPI-ITbM), Nagoya University, Nagoya, Japan; 2 Laboratory of Animal Integrative Physiology, Graduate School of Bioagricultural Sciences, Nagoya University, Nagoya, Japan

**Keywords:** photoperiod, seasonal adaptation, seasonal reproduction, stress response, seasonal affective disorder (SAD)

## Abstract

Organisms that inhabit the temperate zone exhibit various seasonal adaptive behaviors, including reproduction, hibernation, molting, and migration. Day length, known as photoperiod, is the most noise-free and widely used environmental cue that enables animals to anticipate the oncoming seasons and adapt their physiologies accordingly. Although less clear, some human traits also exhibit seasonality, such as birthrate, mood, cognitive brain responses, and various diseases. However, the molecular basis for human seasonality is poorly understood. Herein, we first review the underlying mechanisms of seasonal adaptive strategies of animals, including seasonal reproduction and stress responses during the breeding season. We then briefly summarize our recent discovery of signaling pathways involved in the winter depression–like phenotype in medaka fish. We believe that exploring the regulation of seasonal traits in animal models will provide insight into human seasonality and aid in the understanding of human diseases such as seasonal affective disorder (SAD).

The fitness of a species is determined by how successfully its members pass their genes onto the next generations and maintain the population size. However, from the standpoint of energy demand, reproduction and survival strategies such as thermoregulation are usually in conflict. Survival behaviors deplete resources for reproduction, whereas investment of a significant amount of energy into reproduction compromises individual survival. To cope with dynamic seasonal changes in the environment, seasonal behaviors, including reproduction (1), migration (2), hibernation (3), and molting (1), have evolved as adaptive strategies by which animals adjust their physiologies and behaviors based on the time of year. As a result, annual energy allocation tactics are optimized for the maximum reproductive success while balancing resource investments into seasonally appropriate survival behaviors. To engage in the appropriate adaptive strategies in specific temporal and spatial niches, it is essential for organisms to prepare themselves behaviorally and metabolically long before each season arrives. Therefore, the capacity to precisely predict the coming seasons is crucial for the prosperity of a species. Various environmental factors are used by animals to monitor annual conditions and trigger appropriate physiological responses. For example, increasing ground temperature during spring induces the emergence of ectothermic vertebrates from winter dormancy, leading to subsequent reproduction (4, 5), whereas heavy rainfall is the primary indicator of a suitable breeding time for Eastern Spadefoot toads (6). However, photoperiod is believed to be responsible for regulation of the majority of seasonal behaviors, and the ability of organisms to measure day length in the ambient environment is known as *photoperiodism*. Although the involvement of photoperiod in seasonal adaptations is well established, the underlying molecular mechanism has recently started to be uncovered (7).

In contrast to the clear seasonal responses in animals, the evidence supporting seasonality in humans is more limited (8). However, some seasonal changes are observed in various human physiological processes, including nutrient intake (9), plasma cholesterol (10), blood pressure (11), and vitamin D metabolism (12). Many complex polygenic disorders, such as autoimmune (13), metabolic (14), cardiovascular (15, 16), psychiatric (17), and infectious diseases (18), also exhibit seasonal patterns of incidences. Furthermore, the correlation between seasons and human behaviors is well documented, as exemplified by the annual variation in human conception and death rate (19), violent suicide (20), mood (21), and cognitive brain responses (22). At the molecular level, seasonal variation in the expression of a large set of genes in white blood cells and adipose tissue is reported (23). Nonetheless, it remains unclear how a specific season can exert broad impacts on human physiology.

The remarkable similarity in anatomy and physiology between humans and other vertebrates, particularly mammals, has enabled scientists to explore the mechanisms of various biological functions using animal models and translate the knowledge into humans. In this mini-review, we first review the molecular mechanisms of seasonal adaptive strategies in animals, including seasonal reproduction and self-protective behavior during the breeding season. We then summarize our recent findings on the signaling pathways that might participate in the winter depression–like behavior in medaka fish and a drug screening hit compound, celastrol, that could reverse this depressive phenotype, with the goal of providing cues into the understanding and treatment of human SAD.

## Seasonal Reproduction

Breeding at a specific time of the year, known as seasonal reproduction, represents an adaptive energy distribution tactic by which animals mediate the trade-off between reproduction and survival behaviors. The harsh environment and food scarcity in winter facilitate an energy shift from nonessential functions, including reproduction, to those that are critical for survival, such as thermogenesis. By contrast, climate is moderate and food is abundant in the spring, which is best suited for successful reproduction of parents and survival of offspring. Accordingly, animals usually deliver their young in the spring. Organisms that breed during spring, while the photoperiod is increasing, are long-day (LD) breeders, for example, birds (24), and those reproduce during autumn, with decreasing day length, are short-day (SD) breeders, for example, sheep (25). It only takes a few weeks for a bird egg to hatch, whereas the gestation period for a sheep is approximately 6 months. Therefore, breeding in different seasons ensures the birth and early development of offspring during environmentally optimal spring and early summer in both LD and SD breeders. Seasonal variation in human birthrate is also well documented, and some individuals are thought to be photoresponsive (8), although the underlying mechanism remains obscure.

A rapid and robust response to the changing day length makes Japanese quail (*Coturnix japonica*) an excellent model for exploring photoperiodic signaling pathways. The mediobasal hypothalamus (MBH) has long been believed to be indispensable for seasonal reproduction in quail (26, 27). Initiated by a genome-wide gene expression analysis within the MBH under different light regimes, Yoshimura and colleagues performed a series of pioneering experiments that uncovered the molecular basis for photoperiodic regulation of seasonal reproduction in Japanese quail. Opsin 5 (OPN5)-positive neurons that contact the cerebrospinal fluid in the paraventricular organ are deep-brain photoreceptors that extend fibers to the external zone of the median eminence adjacent to the pars tuberalis (PT) of the pituitary to translate light information into downstream neuroendocrine responses (28). Expression of thyroid-stimulating hormone β subunit (*TSHB*) in the PT is subsequently enhanced by LD stimulus (29). PT-derived TSH (PT-TSH) further induces the expression of deiodinase 2 (*DIO2*) in the ependymal cells (also known as tanycytes) (29) and represses *DIO3* expression. DIO2 is a thyroid hormone (TH)-activating enzyme that converts the prohormone T_4_ to bioactive T_3_, whereas DIO3 metabolizes T_4_ and T_3_ into inactive rT_3_ and T_2_ (30). This reciprocal switching of *DIO2*/*DIO3* expression that locally regulates the concentration of TH within the MBH plays a key role in the regulation of seasonal breeding in quail (31). Several organic anion transporting polypeptides (OATPs) are involved in the transportation of THs in mammals (32). In birds, *OATP1C1* is highly expressed in the ependymal cells within the MBH and choroid plexus, and further functional analysis suggests that it encodes a specific transporter for T_4_ (33). Hence, OATP1C1 could be responsible for the uptake of thyroxine into the MBH that is involved in avian photoperiodic responses. Ultrastructural examination of the quail median eminence, where TH receptors (*THRα*, *THRβ*, and *RXRα*) are expressed (31), reveals morphological differences between gonadotropin-releasing hormone (GnRH) nerve terminals and glial endfeet under different photoperiods (34). Under LD conditions, many GnRH nerve terminals are in close proximity to the basal lamina, whereas under SD conditions the nerve terminals are encased by the endfeet of glial processes and separated from the basal lamina. It is necessary for the hypothalamic neuroendocrine terminals to directly contact the pericapillary space, such as the basal lamina, in order to secrete hormones into the portal capillary (35). Therefore, this dynamic neuroglial plasticity within MBH could modulate seasonal GnRH secretion from the hypothalamus. GnRH then regulates the release of gonadotropins from the anterior pituitary gland, including both luteinizing hormone (LH) and follicle-stimulating hormone (FSH), which subsequently facilitates gonadal development only during the breeding season. It is of note that seasonal plasticity within the GnRH system has also been reported in ewes (36).

**Figure 1.**
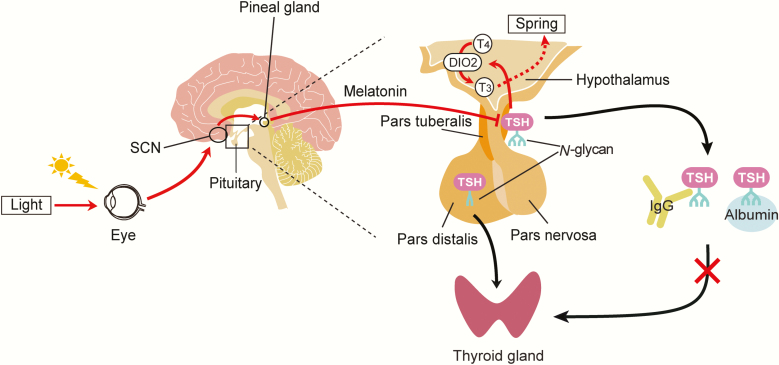
**Pars tuberalis–derived TSH (PT-TSH) is the springtime hormone that regulates seasonal reproduction in mammals.** Light information sensed by the retina in the eye is transmitted to the pineal gland via the suprachiasmatic nucleus (SCN), where the circadian pacemaker is located (7, 37). The pineal melatonin secretion profile with a clear day-night variation reflects photoperiodic information and regulates the production of PT-TSH (37). Long day–induced PT-TSH acts on ependymal cells in the hypothalamus to drive expression of deiodinase 2 (*DIO2*) (37). *DIO2* encodes the TH-activating enzyme that converts the prohormone T_4_ to bioactive T_3,_ thereby transmitting the springtime signal. To avoid functional crosstalk with pars distalis–derived TSH (PD-TSH), which influences metabolism by regulating the hypothalamic–pituitary–thyroid (HPT) axis (42), PT-TSH exhibits a distinct posttranslational glycosylation (41). PD-TSH is modified with sulfated biantennary *N*-glycans and rapidly metabolized in the liver. However, PT-TSH has tissue-specific *N*-glycans and forms a macro-TSH complex with immunoglobulin G (IgG) and albumin in the circulation, resulting in the loss of bioactivity and preventing seasonal thyroid gland overactivity (41). From Nakayama and Yoshimura (2018) (83).

Photoperiodic signal transduction has also been investigated in mammals, revealing both universality and diversity. In mammals, light is sensed by the retina, which then transmits the photoperiodic information to the pineal gland via the circadian pacemaker located in the suprachiasmatic nucleus (SCN) (7, 37) (Fig. 1). Melatonin secreted from the pineal gland exhibits a clear day-night variation, with a peak at night and trace amounts during the day (38). In mammals, unlike birds, melatonin plays an important role in decoding light information, with a longer secretion peak during short days and a shorter peak under LD conditions. Pineal melatonin regulates TSH secretion through receptors in PT (39). Thyrotrophs in the PT do not express TRH and TH receptors; therefore, they are independent from the regulation of the hypothalamic-pituitary-thyroid axis (40, 41). As in birds, PT-TSH also regulates *DIO2*/*DIO3* switching in mammals (37). To avoid functional crosstalk with pars distalis–derived TSH (PD-TSH), which influences metabolism by regulating the hypothalamic-pituitary-thyroid axis (42), PT-TSH employs a distinct posttranslational glycosylation (41). PD-TSH is modified with sulfated biantennary *N*-glycans and rapidly metabolized in the liver. However, PT-TSH contains sialylated multibranched *N*-glycans and forms a macro-TSH complex with immunoglobulin or albumin, resulting in the loss of bioactivity during circulation and preventing stimulation of the thyroid gland (41). Kisspeptin and RFamide–related peptides (RFRPs) are involved in the regulation of seasonal reproduction in mammals. Kisspeptin induces GnRH secretion from GnRH neurons (43), whereas the effects of RFRPs on GnRH neurons vary among species (44-46). To summarize, the photoperiodic TSH-TH signal transduction machinery is widely conserved among vertebrates, but the cells or organs that sense and translate light information are diverse (47).

## Stress Responses During the Breeding Season

Animals activate the hypothalamic-pituitary-adrenal axis within several minutes as a stress response to environmental dangers such as predators, and several lines of evidence demonstrate that the activity of this pathway is elevated during the breeding season as well (48). Nevertheless, how stress responses protect animals from adverse environmental conditions during the breeding season remains obscure. The Japanese medaka fish (*Oryzias latipes*) with clear seasonality is a suitable species to investigate to seek the answer. Transcriptome comparison of medaka hypothalamus and pituitary between LD and SD conditions identified an uncharacterized long noncoding RNA (lncRNA), named *LDAIR* by the authors, as one of the first LD-induced genes; its expression exhibits a robust daily rhythm (49). To clarify gene functions, researchers generated *LDAIR*-knockout medaka using the CRISPR-Cas9 system. The lncRNAs can regulate the expression of a gene neighborhood (50, 51). Consistent with this, 7 genes, including *CRHR2*, in the neighborhood of *LDAIR* were differentially expressed between wild-type and *LDAIR*-null medaka fishes (49). *CRHR2* has been implicated in stress responses in mouse (52, 53). Interestingly, the expression of this gene is photoperiodically regulated in medaka. Further behavior tests demonstrated a stronger stress response of wild-type than the *LDAIR*-null medaka under LD condition, indicating that *LDAIR*-regulated *CRHR2* might also be involved in the stress responses in medaka during the breeding season. It is noteworthy that photoperiodically regulated lncRNAs also function in photomorphogenesis, cotyledon greening, and flowering in plants (54-57). Although lncRNAs share little sequence similarity among species, their roles in seasonal adaptation seem to be conserved.

## Winter Depression–Like Behavior in Medaka

Seasonal rhythms in humans have received renewed interest since the report in 1984 of seasonal affective disorder (SAD), a syndrome characterized by recurrent depressive episodes that occur annually in fall and winter (58). SAD affects 1.4% to 9.7% of the human population in North America, 1.3% to 3.0% in Europe, and 0% to 0.9% in Asia (59). SAD patients usually suffer from depressed mood, disrupted circadian rhythm, social withdrawal, and changes in appetite and body weight (60). Genetic factors, latitudes, and photoperiods are associated with the prevalence of SAD, and a photoperiodic mechanism similar to the one underlying seasonal reproduction has been suggested to be responsible for this syndrome (60). Patients with SAD show decreased photosensitivity in winter, and summer-like bright light treatment is an effective therapy for reversing the symptoms (61, 62). Seasonal changes in color perception have also been observed in healthy humans (63, 64), but their underlying mechanisms remain unknown.

Interestingly, winter depression- and anxiety-like behaviors are widely observed in animals (65), including medaka, which exhibit reduced sociability and increased anxiety-like behaviors under winter conditions (66, 67). Nuptial coloration, in which animals (especially males) undergo rapid color changes and ornamentation, functions as a type of social signaling to attract mates during the breeding season (68). These dynamic changes in body colors emphasize the importance of seasonal regulation of animal vision. The medaka fish develops black stripes, spots, and intense orange-red color on the fins during the breeding season, and thus is a suitable model to explore the phototransduction pathway underlying color perception (69). First, the preference of medaka for orange-red nuptial coloration was tested by presenting fishes with orange-red or gray virtual fish models that were generated by 3D computer graphics (66). Neither winter nor summer medaka showed interest in the gray virtual fish. By contrast, summer medaka were attracted to the orange-red fish model, whereas winter medaka exhibited no such preference, indicating that summer medaka are more sensitive to color. Fish normally swim toward a weak light stimulus (positive phototaxis) but avoid strong light (negative phototaxis) (70). Darkness-induced light-seeking behavior, in which fishes become transiently hyperactive upon loss of illumination, is also common (71). Medaka exhibited both clear phototaxis and darkness-induced light-seeking behaviors under summer conditions, but exhibited neither behavior under conditions simulating winter, proving that light sensitivity is reduced in winter medaka (66). Transcriptome analysis of medaka eye under different photoperiods revealed dynamic changes in the expression of multiple opsin genes as well as genes involved in downstream phototransduction pathways. Furthermore, red cone opsin-null fishes exhibited less phototaxis and weaker preference for the orange-red fish model than wild-type fish under summer conditions, suggesting that summer-induced color perception plays an indispensable role in the emergence of seasonally regulated behaviors (66). Protein synthesis is energetically costly and becomes more challenging in winter. Accordingly, medaka appear to shunt resources from color perception and reproduction to other essential survival functions during winter but reactivate the phototransduction pathway to choose mates during the breeding season. Seasonal changes in opsin gene expression are not rare in animals, and plasticity in color perception is very likely a common adaptive strategy for optimizing energy usage across multiple taxa.

**Figure 2.**
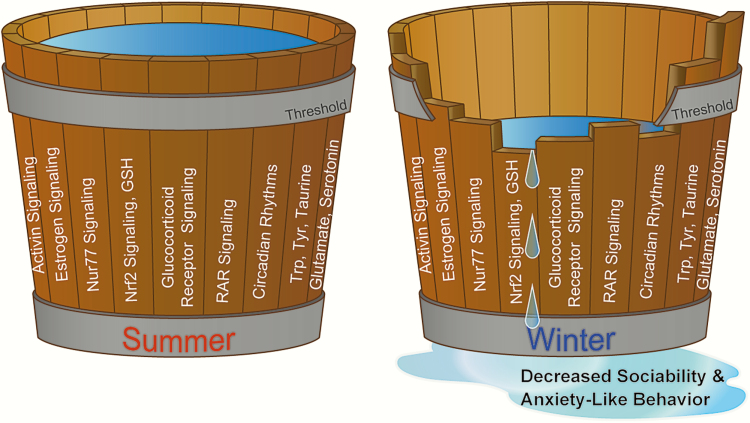
**Involvement of multiple signaling pathways in the emergence of winter depression–like behaviors.** The inactivation of multiple signaling pathways under winter conditions were identified by transcriptomic and metabolomic analyses. A chemical genomics approach revealed that seasonal changes in the NRF2-mediated antioxidant pathways regulate winter depression–like behavior (67).

Although there are obvious differences between fish and humans, many features of their central nervous system are highly conserved, including neurotransmitters, their receptors and transporters, and enzymes related to their synthesis and degradation. Furthermore, antipsychotics affect swimming patterns through conserved pathways (72, 73). Therefore, small teleosts are emerging models for the study of complex brain disorders and are becoming powerful models for pharmacogenetic studies. Most behavioral abnormalities and psychiatric diseases are caused by multiple genes and possibly occur due to dysregulation in multiple brain structures and neural pathways. Therefore, global analyses such as metabolomics and transcriptomics were performed using the whole brain of medaka fish. These analyses revealed seasonal changes in multiple metabolites (eg, serotonin and glutamate), gene expression (eg, circadian clock genes), and signaling pathways (eg, glucocorticoid receptor signaling and estrogen receptor signaling) previously implicated in depression (Fig. 2) (67). To further understand the underlying mechanism of winter depression–like behavior, Nakayama et al employed a chemical genomics approach to screen an existing drug library and identified a NRF2 pathway–activating drug, celastrol, that alleviated the social withdrawal of winter medaka. Treatment with another structurally different NRF2 activator, dimethyl fumarate, also reversed the depression-like behavior in winter medaka. Moreover, *NRF2*-knockout medaka exhibited decreased sociability even under summer conditions (67). All of these findings imply the involvement of NRF2 antioxidant pathway in the regulation of winter depression–like behavior in medaka. Growing evidence suggests the involvement of immune system, such as through inflammatory response and oxidative stress, in the pathophysiology of depression. Depression is known to be accompanied by a decreased levels of the antioxidant glutathione and an increase in the levels of inflammatory markers such as cytokines in the brain of human patients and animal models (74, 75). Since small teleosts have emerged as powerful models for studies of complex brain disorders, these findings may promote the understanding and treatment of SAD in humans. Notably in this regard, mood regulation by light via an melanopsin (OPN4)-dependent mechanism was recently reported in mice (76, 77).

## Conclusions

Seasonal reproduction is a rate-limiting factor for agricultural production. Therefore, genes involved in photoperiodic signaling pathways could emerge as the potential targets to facilitate domestication. In fact, several genes associated with seasonal reproduction have already been implicated in the domestication of various animals, such as the loss of the enzyme activity responsible for melatonin synthesis in most laboratory mouse strains (78-80) and selective sweeps at the *TSHR* locus in domestic chicken (81).

Organisms that share the same habitat experience similar annual changes in the environment, including photoperiod, temperature, and precipitation. It is very likely that animals across multiple taxa, including human lineages, exhibit similar seasonality in physiology and behaviors, such as retinal sensitivity and winter depression. In addition, the underlying molecular basis could also be shared among various species, as exemplified by the aforementioned TSH–TH signal transduction machinery associated with seasonal reproduction and the involvement of NRF2 antioxidant pathway in winter depression in both medaka (67) and humans (82). Despite the substantial differences between humans and other vertebrates, many biological functions are conserved, making animal models invaluable tools for the scientific community. Therefore, elucidating the mechanism of seasonal adaptation in animals will contribute to our understanding of human seasonality.

## Abbreviations

DIO2: deiodinase 2
FSH: follicle-stimulating hormone
GnRH: gonadotropin-releasing hormone
LD: long-day
LH: luteinizing hormone
lncRNA: long noncoding RNA
MBH: mediobasal hypothalamus
OATP: organic anion transporting polypeptide
PD-TSH: pars distalis–derived TSH
PT: pars tuberalis
PT-TSH: pars tuberalis–derived TSH
RFRP: RFamide–related peptide
SAD: seasonal affective disorder
SD: short-day
T2: diiodothyronine
T3: triiodothyronine
T4: thyroxine
TH: thyroid hormone
TSH: thyrotropin (thyroid-stimulating hormone)

## Additional Information

***Disclosure Summary:*** The authors report no conflict of interest.

***Data Availability:*** Data sharing is not applicable to this article as no datasets were generated or analyzed during the current study.
